# Financial InceNtives for cArdiac rehabilitatioN ComplEtion (FINANCE) (single blind pragmatic RCT)

**DOI:** 10.1097/MD.0000000000032936

**Published:** 2023-02-22

**Authors:** Jae In Lee, Jae-Young Han, Hae-Bin Gwak, Chang-Won Moon, Min Kyun Sohn, Sungju Jee, Chul Kim

**Affiliations:** a Department of Rehabilitation Medicine, Chungnam National University Hospital, Chungnam National University College of Medicine, Daejeon, Republic of Korea; b Department of Physical Medicine and Rehabilitation, Chonnam National University Hospital, Chonnam National University Medical School, Gwangju, Republic of Korea; c Department of Rehabilitation Medicine, Inje University Sanggye Paik Hospital, Seoul, Republic of Korea.

**Keywords:** cardiac rehabilitation, completion rate, incentive, participation rate

## Abstract

**Introduction:**

Cardiac rehabilitation (CR) is strongly indicated in patients with acute myocardial infarction (MI), and has been proven to reduce mortality and recurrence and improve patients quality of life. Although clinical guidelines for CR have already been developed domestically and internationally, hospital-based CR remains underutilized. Currently, studies exploring strategies to improve CR participation in South Korea and Asia are limited.

**Objectives:**

This study aims to compare the effect of providing CR financial incentives to post-MI patients referred for outpatient CR and to confirm the effect of increasing CR participation and completion rates.

**Methods:**

This single-blind, pragmatic, randomized controlled trial will be conducted at 2 tertiary hospitals for CR after acute MI. The control and experimental groups will be randomized, with each group consisting of 24 participants (total of 48 participants) assigned in a 1:1 ratio. The experimental group will receive 4, 7, and 11 USD per completed session of CR during the 1st to 12th, 13 to 24th, and 25th to 36th sessions of CR, respectively, for 3 months after enrollment. Participants who completed the 36 sessions will receive 260 USD incentives. The primary outcomes at 3 months will be used to assess the CR participation rate, as the number of CR sessions completed, and CR completion, as attendance of sessions greater than 50%, thus completion of ≥18 sessions. The outcomes will be used to compare changes in cardiorespiratory function (VO_2_ max, VO_2_ at anabolic threshold), the Korean activity scale index, EuroQol 5 dimensions, and the patient health questionnaire at 3 months after discharge and 6 and 12 months after baseline.

**Discussion:**

Providing financial incentives may confirm the effect of increasing CR on participation and completion rates.

## 1. Introduction

Cardiac rehabilitation (CR), which consists of supervised exercise programs, patient education, risk factor management, and stress control, is a customized intervention for patients with cardiovascular disease. CR has been most strongly indicated in patients with acute myocardial infarction (MI),^[1]^ and various studies have demonstrated its effect in reducing mortality, recurrence, and hospital readmissions while improving exercise capacity and quality of life.^[2–4]^

Although strong recommendations and benefits of CR have been emphasized in the literature, the actual participation rate in CR remains low worldwide. Among CR-eligible Medicare beneficiaries in the United States, only 24.4% participated in CR, of whom 26.9% completed the CR program.^[5]^ In Japan, CR participation rates were estimated to be as low as 3.8% to 7.6%.^[6]^ Globally, approximately 38.8% to 54.7% of countries have provided CR,^[6,7]^ but CR participation remains at approximately 30% to 40% worldwide.^[6,8]^

According to a global CR density report published in 2019, the CR density in South Korea was 22, ranking 27th out of 111 countries with CR programs.^[9]^ A national study reported CR density as approximately 10 AMI incidences/capacity/year, indicating that 10-fold more CR institutions are required for the yearly AMI incidence, with only 1.5% of acute myocardial infarction patients (960/64,982) participating in outpatient CR after discharge. Thus, despite CR coverage by the National Health Insurance, hospital-based CR remains underutilized in South Korea.^[10]^ Moreover, despite enrollment in outpatient CR starting at 47%, adherence to the program has decreased to 17% in regional cardiocerebrovascular centers in South Korea.^[11]^ Surveys of the medical staff in these centers indicated that time, distance, and transportation issues were relevant barriers to CR, followed by cost burdens. Additionally, a larger study questioning CR attenders compared with non-attenders indicated that patients identified logistical factors, such as distance, cost, and transportation problems, as major barriers to center-based CR.^[12]^ A systematic review analyzing studies of CR participation in the USA and Europe reported that lack of transportation or distance from CR facilities were important factors for low CR adherence.^[13]^

Various intervention strategies have been implemented worldwide to increase participation and adherence to CR. A previous study by Gaalema et al^[14]^ reported a significant increase in CR participation with doubling of CR completion in patients with low socioeconomic status in the US by providing financial incentives to CR-eligible patients. Currently, there are insufficient studies exploring strategies to improve CR participation in South Korea and in Asia, in general.

We hypothesized that issues of transportation difficulties, cost burden, and time could be alleviated by providing financial incentives. Therefore, this study examines the efficacy of financial incentives as transportation aids for increasing CR participation and completion among acute MI patients in South Korea.

## 2. Methods

### 2.1. Study design and protocol registration

This study is a single-blind, pragmatic, randomized control trial with an allocation ratio of 1:1, implemented until June 2023. This randomized control trial was registered at the Clinical Research Information Service (CRIS, WHO International Clinical Trials Registry Platform as the 11th member of the Primary Registry: Registration No: KCT0006063). This protocol was developed based on the guidelines of the Standard Protocol Items: Recommendations for Interventional Trials (SPIRIT, https://www.spirit-statement.org/).

### 2.2. Study setting

Patients at 2 tertiary hospitals, Chungnam National University Hospital and Chonnam National University Hospital, will be screened for inclusion.

### 2.3. Subjects and recruitment

Among the patients who visited the hospital for outpatient CR, the study objectives will be explained and patients who provided consent will be selected and recruited. Separate recruitment notices will be posted in the therapy rooms of the rehabilitation center and outpatient clinics of the Department of Rehabilitation Medicine and Department of Cardiology. Banners for recruitment will be installed at the entrance of the hospital’s main building.

#### 2.3.1. Selection criteria.

- Male and female patients aged between 19 and 85 years.
- Patients hospitalized for acute MI and referred for outpatient CR after acute treatment and discharge.
- Patients who agreed to participate in CR included as research subjects.

#### 2.3.2. Exclusion criteria.

- Patients contraindicated for cardiorespiratory exercise-stress testing and exercise training or those who had difficulty participating in this study because of other medical reasons and discretion by the researcher.
- Contraindications included unstable angina symptoms, acute myocardial infarction (within 2 days), uncontrolled ventricular arrhythmias, 3rd-degree atrioventricular block without a pacemaker, acute congestive heart failure, severe aortic stenosis, acute systemic disease, thrombophlebitis or intracardiac thrombosis, active or suspected myocarditis, and pericarditis.
- Patients determined to be at risk of serious health problems because of exercise training.

### 2.4. Binding and allocation

The control and experimental groups will be randomized, with each group having a 1:1 assignment ratio. Group assignments will be determined using computer-based random number generators and will be known only by the research assistant after opening a sealed envelope containing a computer-generated sequence sheet.

The participants will know which group they belong to; however, the assessor will not be aware of each participant’s group, and the participants will be instructed not to inform the assessor. The person managing the data will not know the subject group until the analysis of the study is complete. If any participant is included in the study discontinuation criteria, the principal investigator will reveal the blinding and progress to standard treatment.

### 2.5. Sample size

The sample size was based on the results of a study on providing incentives for CR (e.g., session-completion rate:55.4% vs 29.2%, *P* = .002).^[14]^ However, considering the low CR completion rate in Korea, the session-completion rate was revised to 55.4% versus 15.4% (*P* = .002), and the sample size power was 0.8, thereby requiring 22 subjects for each group. Additionally, considering a 10% dropout rate, 24 participants will be required for each group, resulting in 48 participants. Twenty-four participants (12 each in each group per hospital) will be recruited because this study will be conducted at 2 institutions.

### 2.6. Intervention method

#### 2.6.1. Experimental group.

The participants will participate in 36 outpatient rehabilitation exercise programs with incentives. Three CR sessions will be conducted weekly. The experimental group will receive 5000 (approx. 4 USD), 10,000 (7 USD), and 15,000 Korean Won (11 USD) per completed session of CR during the 1st to 12th, 13 to 24th, and 25th to 36th CR sessions, respectively. Participants who will complete the 36 sessions will receive ₩ 360,000 (260 USD) incentives.

#### 2.6.2. Control group.

The participants will participate in the same 36 sessions of outpatient rehabilitation exercise programs as those in the experimental group. However, the participants randomized to the control group will not receive incentives.

#### 2.6.3. Exercise program for both groups.

The CR program will consist of 36 outpatient sessions conducted over approximately 12 weeks. Each exercise session will consist of warm-up exercises (5–10 minutes), main exercises (30 minutes), and finishing stretching exercises (5–10 minutes). The main exercise will consist of aerobic exercise through a treadmill or ergometer under supervision, with the goal of reaching an exercise intensity of 70% to 85% of the baseline maximal heart rate. Drug compliance monitoring, appropriate nutrition, risk factor management, benefits of CR, and exercise education will be included.

#### 2.6.4. Adherence plan for participants.

All participants will be provided with a Galaxy Fit2(42 USD) Bluetooth Fitness Tracking Smart band after completing the assessment at 3 months to help them maintain self-care after completion of the CR program. All expenses of the follow-up assessments at baseline and 3, 6, and 12 months will be supported by funds provided by the Korea National Institute of Health, Korea Disease Control, and Prevention Agency, Cardiovascular Disease Department.

### 2.7. Study discontinuation criteria

Subjects who drop out or discontinue prior to the completion of the study are indicated as follows:

- When the participant withdraws consent to participate in the trial.
- Violation of the selection criteria or falling under exclusion criteria.
- When a researcher or clinical trial subject violates the study plan.
- When a severe adverse event occurs in a clinical trial (e.g., hypotension, chest pain, and loss of consciousness).
- If the researcher or clinical trial subject determines that it is necessary to terminate participation because of an adverse event.
- Cases of disease recurrence.
- If no data have been collected, because the study has never been assessed since randomization and when the subject cannot be tracked.
- If it is determined that it is unreasonable to continue a clinical trial while the study is in progress.

### 2.8. Data collection and analysis

#### 2.8.1. Assessment timing and assessment criteria.

**Figure 1.**
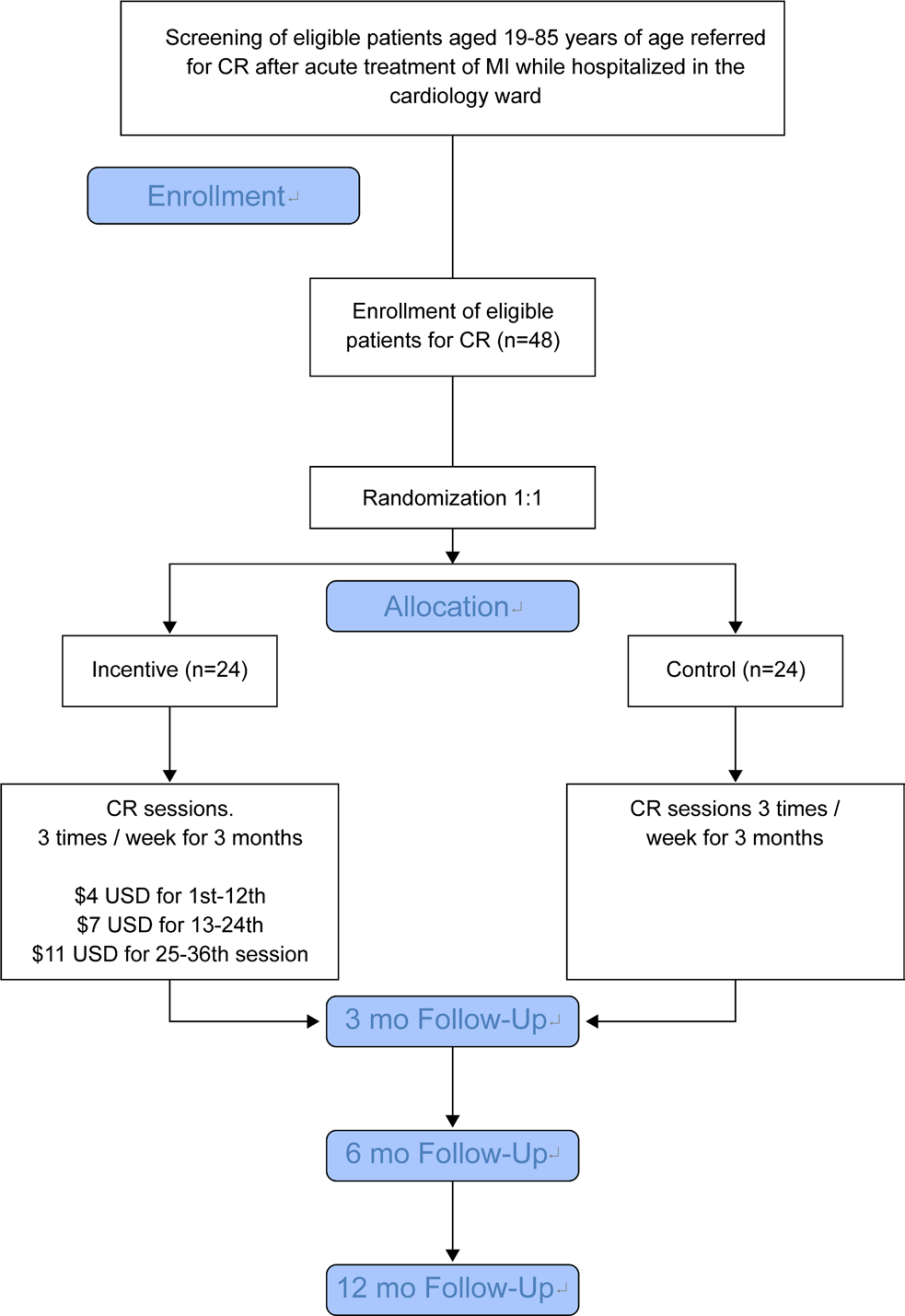
Flowchart of the data collection.

All participants will complete baseline assessments and chart review (after consent), followed by assessments at 3 months after discharge (after the exercise program), and at 6 and 12 months after baseline evaluation (follow-up assessment) (Fig. 1).

During baseline assessment, basic demographic information (sex, age, body mass index, occupation, distance/time from home to the hospital, insurance type) and clinical information [cardiac arrest type, diagnosis, number of coronary artery stenosis/occlusion, intervention type/result, left ventricular ejection fraction (LVEF) (%), remaining coronary occlusion (%), lipid/glucose profile, echocardiogram findings, risk factor information (diabetes mellitus, hypertension, dyslipidemia, obesity), history of cardiocerebrovascular disease, laboratory results, body composition analysis and physical measurement, cardiopulmonary exercise testing (CPET) results, lifestyle risk factors (smoking, drinking, physical activity)] will be obtained through chart review. Additionally, basic information, patient-reported outcome measures (PROM), and clinical outcomes will be assessed.

At 3 months (assessment after completion of the exercise program), data will be collected on exercise therapy completion rate and the number of session participations.

At 3 and 6 months, body composition analysis, physical measurements, CPET results, PROM, and clinical outcomes will be assessed.

At 12 months, through chart review, echocardiography, laboratory test results, and medical information, if available, will be recorded, and body composition analysis and physical measurements, CPET results, PROM, and clinical outcomes will be assessed.

Patients who discontinue participation in the assessments during the study course will have values used in the analysis until the dropout period. All patients were sent an identical reminder text message informing the participants of the date, time, and location of the upcoming evaluation to promote patient retention and follow-up.

Table 1 presents the details of the data collection.

**Table 1:** Schedule of enrollment, intervention, and assessments.

Timeline assessment item		① Consent	② Baseline	③ Exercise end point 3 months	④ 6 months	⑤ 12 months
Screening		√				
Enroll *		√				
Chart review †	Basic information		√			
	Occupation		√			
	Past history		√			
	Heart disease information		√			
	Cardiovascular imaging and detailed PCI Information		√			
	Echocardiography ‡		√			√
	Lab §		√			√
	Current medication		√			
	Cardiac rehabilitation information		√			
	KASI prior to the onset		√			
Intervention	Incentive		→			
Subject questionnaire (Assessment Index) ∥	Number of cardiac rehabilitation participation ¶			√		
	Body composition analysis and physical measurement #		√	√	√	√
	CPET results **		√	√	√	√
	KASI, EQ-5D, PHQ-9, and other PROM ††		√	√	√	√
	Clinical outcome §§		√	√	√	√
	Patient questionnaire ∥∥			√		√

CPET = cardiopulmonary exercise testing, EQ-5D = EuroQol 5 dimensions, KASI = Korean activity scale index, PCI = percutaneous coronary intervention, PHQ-9 = patient health questionnaire, PROM = patient-reported outcome measures, VO_2_ = oxygen consumption rate.

* Assignment of research number in order after obtaining consent.

† Chart review through medical records.

‡ Baseline: Preparation of initial trial results during hospitalization, 12-month results recorded if available in the medical record.

§ Baseline: Preparation of initial trial results during hospitalization, 12-month results recorded if available in the medical record.

∥ Questionnaires filled out by actual subjects.

¶ Record of participation in exercise program 36 times.

# Physical measurement: participation prior to exercise stress testing (including weight, BMI, etc).

** Exercise stress testing (including CPET test, VO_2_ peak, VO2 at anabolic threshold etc).

†† Subject questionnaire for PROM (KASI, physical activity, EQ-5D, and PHQ-9).

§§ Subject questionnaire for clinical outcome (including return to work, emergency room visits, and rehospitalization).

∥

∥ At the end of the 3rd and 12th months, the satisfaction assessment of the cardiac rehabilitation program is conducted through patient questionnaire.

### 2.9. Outcome evaluation

#### 2.9.1. Primary outcome.

The primary outcomes at 3 months will be the CR participation rate, defined as the number of CR sessions completed, and the CR completion rate (attendance of sessions greater than 50%; thus, completion of ≥ 18 sessions). Changes in PROM functional status using the Korean activity scale index (KASI), quality of life assessments in EuroQol 5 dimensions, and depression evaluation using the patient health questionnaire will be compared from baseline to 3, 6, and 12 months.

#### 2.9.2. Secondary outcome.

The secondary outcomes at 3 months will be used to assess changes in cardiopulmonary endurance by 1 MET (peak oxygen consumption rate [VO_2_] 3.5 mL/kg/minutes), VO_2_ max, and VO_2_ at the anabolic threshold. At 6 and 12 months, improvement and maintenance of cardiorespiratory endurance by 1 MET (peak VO_2_ 3.5 mL/kg/minutes), VO_2_ max, and VO_2_ at anabolic threshold will be assessed.

#### 2.9.3. Benefits and risks for participants.

##### 2.9.3.1. Benefits.

Participation in this study does not guarantee medical benefits. However, incentives motivate people to increase their participation in CR exercises, and participants can improve their recovery from the disease by actively participating in the program.

##### 2.9.3.2. Risks and discomforts.

Regarding participation, a difference exists only in the provision of incentives. However, there are no additional risks or discomfort for participating in the study because all procedures are to be performed in accordance with standard practice procedures.

### 2.10. Monitoring

#### 2.10.1. Standard medical procedures and research procedures.

The interventions used in this study will differ in the provision of transportation expenses between the control and experimental groups; otherwise, there will be no difference in treatment compared to conventional treatments. Exercise stress testing, cardiovascular imaging, labs, and echocardiography are standard practice procedures in which the cost of the test will be supported by research funds.

#### 2.10.2. Continuous safety monitoring and data safety monitoring plans.

The principal investigator, project manager, and monitoring agent from the Korean Center for Disease Control and Prevention will conduct monitoring every 6 months. If a severe adverse reaction occurs during the study, the event will be immediately reported to the Institutional Review Board (IRB). If a minor adverse reaction occurs, the event will be reported to the IRB after monitoring is performed by the principal investigator and project manager.

Interim analyses and reports will be reported once a year to the IRB, and if modifications are required for the study protocol, permission will be granted by the IRB.

Auditing will be performed by the IRB once a year or when severe adverse events, unexpected events, or significant protocol deviations are reported, and a decision on whether to terminate the study early will be taken.

#### 2.10.3. Data-monitoring committee (DMC), safety monitoring methods, and cycles.

The DMC will be comprised of several members of the IRB, including at least 1 outside the hospital. When a participant receives an explanation and consent form, the principal investigator will explain the reporting system of the adverse reactions and instruct the participant to immediately inform the researcher if an adverse reaction occurs. The presence or absence of adverse reactions will be confirmed upon completion of the assessment. DMC will analyze the study accruals and report adverse reactions every 4 months or when severe adverse events or unexpected events are reported.

#### 2.10.4. Adverse drug reaction report, noncompliance with studies, and unexpected event report.

Severe adverse events, noncompliance with major studies, or unexpected problems will be reported to the IRB by monitoring personnel within 15 working days from the date of recognition by the researcher. If the event is death or life-threatening, it will be reported to the IRB within 7 days.

### 2.11. Statistical analysis

The analysis of this study was performed using an intention-to-treat and per-protocol method. CR participation, defined as the number of sessions completed, will be analyzed as a continuous variable using the student *t* test or Mann–Whitney *U* test, based on the normal distribution of the data. CR completion and categorical variables will be analyzed using chi-square or Fisher’s exact test. Missing data will be replaced using the last observation carried forward or the mean value. In the case of more than 20% of dropouts, missing data in the intent-to-treat analysis will be replaced using multiple imputations. The intention-to-treat population will include all enrolled participants, and the per-protocol analysis will include patients who have completed the 3-month evaluation.

Changes in secondary outcomes over time for the CPET, KASI, EuroQol 5 dimensions, and patient health questionnaire will be analyzed using paired t-tests with comparisons from baseline. Analyses will be conducted for each of the intervention, control, CR completion, and non-completed groups. For non-normal distributions, the Wilcoxon signed-rank test will be used.

Statistical significance will be set at *P* < .05, and SPSS version 20 (IBM SPSS Statistics, New York, NY) will be used for the statistical analysis.

### 2.12. Provisions for post-trial care

The participants will be regularly followed-up in the outpatient clinic of the Department of Rehabilitation Medicine. Adverse events or worsening of symptoms will be evaluated and monitored by physicians and annual CPET examinations.

### 2.13. Ethical approval and consent to participate

Approval from the IRB was obtained prospectively before conducting the research, and the board agreed to confirm the implementation and process of the research. Ethical approval was obtained from the Research Ethics Committees of Chungnam National University Hospital (CNUH 2021-01-025) and Chonnam National University Hospital (CHUH-2021-100) through peer reviews.

#### 2.13.1. Consent process of participants.

The objectives and methods of this clinical study will be explained to the participants through an explanatory text, and only subjects who know the objectives of the study and have a written consent form will participate in the study. The records identifying the subjects will be kept confidential, and the identity of the subjects will remain confidential if the results of the clinical study are published. Consent will be obtained by specifying and explaining that the subjects may discontinue participation at any time during the research period. The principal investigator and the patient physician will fully explain the study to the participants and obtain written consent. Only the participants will be able to provide consent, and they will be given sufficient time to think and make their own decisions. The researcher or physician will not encourage participants to provide consent, and if necessary, the participants will be given time to go home, think over, and sign the consent form.

#### 2.13.2. Protocol amendments.

Interim analysis and reports will be provided once a year to the IRB, and if modifications are required for the study protocol, permission will be granted by the IRB and it will be reported to the trial registry. In the event of changes in the study protocol or changes based on DMC reports and adverse reaction analysis, such changes will be reported, and corresponding permission will be received from the IRB or the Korea Disease Control and Prevention Agency.

### 2.14. Privacy measures for participants

#### 2.14.1. Storage method and storage period.

The medical records of the patients (registration number) and pathology numbers will be kept in a separate file under the responsibility of the researcher-in-chief. Each patient will receive a unique code number so that the identification of personal information is prohibited through the research data. The data will be entered into a spreadsheet and stored in a locked cabinet. According to Article 15 of the Enforcement Rules of the Bioethics and Safety Act in Korea, records related to research will be preserved for 3 years from the date of research completion.

#### 2.14.2. Destruction method.

Documents containing personal information that pass the 3-year retention period will be destroyed in accordance with the relevant laws and regulations. Electronic files will be permanently deleted. Paper records such as printed materials and signatures will be shredded or incinerated. Documents will be destroyed in accordance with Article 16 of the Enforcement Decree of Personal Information Protection.

## 3. Discussion

CR is an essential treatment process with proven safety and effectiveness in improving exercise capacity, quality of life, and prognosis of patients with cardiovascular disease, and is currently being implemented in 111 countries worldwide. Scotland (SIGN: The Scottish Intercollegiate Guidelines Network),^[15]^ the UK (NICE: National Institute for Health and Clinical Excellence),^[16]^ the U.S. (AHA: American Heart Association),^[17]^ Europe (ESC: European Society of Cardiology),^[18]^ Canada (CACR: Canadian Association of CR),^[19]^ and Japan (JCS: Japanese Circulation Society)^[20]^ have already published clinical practice guidelines for CR with a high level of evidence and strong recommendations. In Korea, clinical practice guidelines for CR^[21]^ were developed with the support of the Health and Medical Research and Development Project of the Korea Health Industry Development Institute and are distributed to related academic societies and cardiovascular hospitals.

However, because of various factors that impede participation in CR, the actual participation rate among patients in need of CR remains low, at approximately 30% to 40%, even in medically advanced countries.^[6]^ Each country is striving to increase its participation rate in cardiac rehabilitation. Conversely, Korea reports even lower levels because of reasons such as lack of awareness regarding the concept and importance of CR by physicians and patients; lack of cardiac rehabilitation specialists and facilities; and logistic barriers, such as distance, time, and cost. Currently, cardiac rehabilitation programs are implemented in approximately 40 university hospitals and cardiovascular hospitals nationwide, including 11 regional cardiocerebrovascular disease centers. Korea has a considerably lower level of 5%, which is significantly lower than that of the aforementioned medically advanced countries. Moreover, despite the implementation of cardiac rehabilitation health insurance benefits initiated in February 2017, the statistics show that only 26 hospitals filed claims for CR health insurance in 2018, suggesting that CR has not yet been properly implemented in Korea.^[10]^ Although clinical practice guidelines for CR have already been developed domestically and internationally, the implementation practice rate and outcomes, such as dissemination and distribution of the guidelines in hospital clinical sites, are insufficient.

This study is for patients who were referred for CR after hospitalization in the cardiology department for acute MI. This study aims to compare the effectiveness of offering CR incentives to CR-eligible patients with traditional CR. Therefore, additional efforts to increase CR participation in Korea are urgently required. This study has several strengths. This is the first Asian study to assess the effect of financial incentives on increasing CR participation and completion. The results may help establish more efficient guidelines for the national CR program and improve CR participation. Such effects may help reduce the recurrence of MI, improve patients quality of life, and increase the rate of return to the social workplace.

This study has limitations. It does not consider economic status in the selection process of the subjects. The effect of financial incentives may be emphasized in a patient population with low economic status; however, in Korean healthcare, such a population already receives financial support from the National Healthcare Insurance policy. Another limitation is that this study will be centered on tertiary hospitals; thus, it is expected that it may be difficult to apply the research conclusions to local regional clinics. However, the results of this study will be utilized efficiently in establishing effective CR programs in South Korea, because the national CR guidelines are based on tertiary hospital settings.

This study is supported by a grant from the Department of Cardiovascular Disease, Korea National Institute of Health, Korea Disease Control and Prevention Agency (Grant No. 2020ER630500). The data and results will be reported to the Korean National Institute of Health and submitted for publication in peer-reviewed journals and international conferences.

## 4. Conclusion

This study aims to compare the effect of providing CR financial incentives to post-MI patients referred for and eligible for outpatient CR and to confirm the effect of increasing the participation rate and completion rate of CR. Future studies investigating specific barriers to CR in Asian populations, especially South Korea, may help alleviate the low CR participation rate and emphasize the results of this study. The data and results can be used to improve and design new health program guidelines for cardiovascular rehabilitation.

## Author contributions

**Data curation:** Hae-bin Kwak, Jae In Lee.

**Funding acquisition:** Chul Kim.

**Investigation:** Jae In Lee, Sungju Jee, Jae Young Han, Chul Kim.

**Resources:** Jae In Lee, Chang-won Moon, Min Kyun Sohn.

**Supervision:** Sungju Jee, Jae Young Han, Chul Kim.

**Writing – original draft:** Jae In Lee.

**Writing – review & editing:** Jae In Lee, Sungju Jee.
